# Development of Methods for Specific Capture of Biological Targets on Aluminum Substrates: Application to *Bacillus subtilis* Spore Detection as a Model for Anthrax

**DOI:** 10.3390/s22093441

**Published:** 2022-04-30

**Authors:** Ethan P. Luta, Benjamin L. Miller

**Affiliations:** Department of Dermatology, University of Rochester, Rochester, NY 14642, USA; ethan_luta@urmc.rochester.edu

**Keywords:** silanes, alumina, surface functionalization, anthrax

## Abstract

Many (if not most) biosensors rely on functional silane coatings as a first step toward covalent immobilization of specific capture molecules. While methods for silanization of silica (SiO_2_) surfaces are very well developed, less has been done to develop and characterize silanization methods for alternative substrates, such as alumina (Al_2_O_3_). In particular, the behavior of Al_2_O_3_ coatings grown on aluminum under ambient conditions has not been studied. To address this issue, we have tested solution-phase deposition of two silanes on Al_2_O_3_ (3-aminopropyl triethoxysilane and 3-triethoxysilyl)propylsuccinic anhydride) and their applicability to analyte-specific biosensing. Contact angle measurements and imaging via Scanning Electron Microsopy (SEM) were employed to characterize surfaces. We find that 3-aminopropyl triethoxysilane produces well-behaved films and demonstrate that this surface can undergo further reaction with glutaraldehyde followed by an anti-*Bacillus subtilis* antibody to yield functionalized Al_2_O_3_ surfaces capable of specific capture of *B. subtilis* spores (a model of *B. anthracis*, the causative organism of Anthrax). In contrast, 3-triethoxysilyl)propylsuccinic anhydride did not behave well with Al/Al_2_O_3_ under the reaction conditions tested. In addition to providing specific protocols for Al/Al_2_O_3_ functionalization, this work highlights the importance of surface chemistry assessment in the development of new sensors.

## 1. Introduction

There is a continuing need for new methods for the detection of pathogenic organisms [1,2,3,4]. While extensive research effort on pathogen detection has been expended in the sensing field, optical and electrical sensing methods able to provide increased speed, sensitivity, and selectivity at low cost are desirable [5,6,7]. In the context of inexpensive electrical or optical sensors, aluminum is particularly attractive as a base material for new device development [8,9]. Aluminum is an abundant material, and its extensive commercial use has led to the development of manufacturing-scale methods for its deposition in a broad range of formats. However, for aluminum to be useful as the starting point for the production of sensors, methods must be available for reliable, activity-preserving covalent attachment of biomolecules used for specific analyte capture to its surface.

On exposure to oxygen, including in ambient atmosphere, Al metal forms a thin (1–1.5 nm) surface layer of aluminum oxide (Al_2_O_3_, or alumina) [10,11,12]. Porous anodic alumina has been studied extensively as a substrate for sensor production, and requisite methods for immobilization of capture molecules on porous alumina are well developed [13]. Likewise, silanization of Al_2_O_3_ nanostructures (a typical precursor to biomolecule immobilization) has been studied [14,15]. In contrast, as far as we are aware, there are no studies available regarding the immobilization of capture biomolecules on aluminum metal substrates with ambient Al_2_O_3_ as a precursor to their use as biosensors. We sought to address this gap, and in particular to assess (1) whether the thin layer of ambient Al_2_O_3_ would be sufficiently robust to allow for functionalization, and (2) whether antibodies covalently attached to such substrates via methods we have previously developed for Si/SiO_2_ retained their analyte binding activity and specificity. Here, we describe successful attachment of antibodies specific for *Bacillus subtilis* (a commonly used, safe to handle surrogate for *Bacillus anthracis*, the causative agent of Anthrax) [16,17] spores to Al/Al_2_O_3_ substrates and specific detection of spores as visualized via scanning electron microscopy (SEM). *B. anthracis* remains a pathogen of particular concern, primarily because of its potential as a biowarfare agent [18]. Thus, sensors and diagnostics able to detect *B. anthracis* spores are the subject of considerable continuing research [19,20], leading us to choose this pathogen as a model system.

## 2. Methods

### 2.1. Materials

15 × 15 mm substrates consisting of an aluminum layer deposited on glass (SiO_2_) were obtained from BiSenTech, Inc. (Rochester, NY, USA). The aluminum layer is covered with approximately 1.0 to 1.5 nm of aluminum oxide (Al_2_O_3_). Toluene was purchased from Fisher chemical (Hampton, NH, USA) (Cat. T324-4). 3-Aminopropyl triethoxysilane and 3-triethoxysilyl)propylsuccinic anhydride were purchased from Gelest, Inc. (Morrisville, PA, USA). *Bacillus subtilis* spores were obtained from Sigma Aldrich (Saint Louis, MO, USA) (Cat. 110649). Anti-*Bacillus subtilis* was obtained from MyBioSource (San Diego, CA, USA) (Cat. #MBS612878); anti-human interleukin-6 (IL-6) was obtained from Biolegend, (San Diego, CA. USA) (Cat. 501110).

### 2.2. Substrate Functionalization

The thin layer of Al_2_O_3_ produced under ambient conditions on aluminum is too fragile to permit cleaning with caustic solutions such as piranha (H_2_SO_4_/H_2_O_2_). Instead, prior to silanization, substrates were washed with 100% ethanol, followed by 100% isopropanol, followed by distilled, deionized water (ddH_2_O). This wash procedure was repeated 3 times sequentially. The substrates were then dried with a stream of nitrogen, and UV-Ozone treated for 30 min (UV-Ozone cleaner: Procleaner^™^, Bioforce nanoscience, Virginia Beach, VA, USA). This step was taken because Sun et al. have shown that exposure of Al_2_O_3_ to UV-Ozone cleaners increases the ratio of Al-OH, required for reaction with silanes, within the oxide and decreases the ratio of Al-O-Al groups [21].

### 2.3. Silanization

After UV/Ozone treatment, substrates were placed in a 1% solution of either 3-aminopropyltriethoxysilane (APTES) or (3-triethoxysilyl)propylsuccinic anhydride (TESP-SA) in dry toluene (*v/v*; toluene dried via distillation over Na metal under N_2_) for 30 min at ambient temperature (21–22 °C) with shaking on a rotating platform. The substrates were then rinsed in dry toluene for 5 min, dried with a stream of nitrogen and then finally cured in an oven at 110 °C for 1 h.

### 2.4. Glutaraldehyde and Antibody Functionalization

APTES-functionalized Al/Al_2_O_3_ substrates intended for antibody deposition were placed in an 8% (*w/w*) glutaraldehyde solution in water for 30 min. The chips were then washed three times in 1× PBS, pH 7.5, for 5 min in each solution. The substrates were then transferred to a solution of 100 µg/mL anti-*Bacillus subtilis* antibody in 1× PBS, pH 7.5, or 100 μg/mL of anti-IL-6 in PBS solution pH 7.5, to be used as a negative control. Substrates were allowed to react with the respective antibody solutions for 30 min and were then washed 3 times in washing buffer consisting of 150 mM NaCl, 10 Na_2_HPO_4_, 10 NaH_2_PO_4_-2H_2_O, 3 mM EDTA, and 0.005% TWEEN-20.

### 2.5. Target Incubation

The substrates were transferred to a solution of *Bacillus subtilis* spores (Sigma Aldrich, 110649) at a concentration of 10^7^ CFU/mL for 2 h. In order to test the “worst case scenario” for discriminating specific, antibody-mediated spore capture vs. nonspecific adhesion of spores, substrates were not blocked prior to their incubation with the spore solution. Following spore exposure, substrates were then washed in washing buffer, followed by water, and finally dried with nitrogen. These substrates served as experimental substrates to assess the specific binding of antibodies to *Bacillus subtilis* spores in solution. Unfunctionalized substrates as well as substrates functionalized with APTES alone or APTES followed by glutaraldehyde served as a control for non-specific spore binding to a chemically functionalized surface.

### 2.6. Contact Angle Measurements

Contact angles were measured using a Ramé-hart Model 100-00-115, NRL C.A Goniometer (Mountain Lake, NJ, USA). Bare Si/SiO_2_ and Al/Al_2_O_3_ substrates were treated with UV/ozone plasma prior to evaluation; functionalized substrates were tested immediately after silane deposition. For each measurement, the horizontal line (stationary arm) was aligned parallel to the surface of the substrate. A single water droplet was dispensed on the substrate surface. The height of the stage was adjusted such that the horizontal marker line in the goniometer window aligned to the droplet−surface interface. The stage was then moved so that the fulcrum (intersection) of the stationary and moving arm was at the outermost (right side) point of contact between the droplet and the surface. The moving arm was then adjusted to measure the contact angle. The stage was moved to the left side of the droplet and the measurement was repeated. These steps were repeated for three separate droplets, the results of which were averaged and the standard deviations calculated.

### 2.7. SEM Analysis

Scanning electron microscopy (SEM) was conducted using a Zeiss Auriga Scanning Electron Microscope (Oberkochen, Germany). Images were obtained using the secondary electron detector, and at 20 kV acceleration voltage, using the standard 30 µm aperture. To initiate analysis, the edge of the chip was first located. The image was then focused, and stigmation and aperture alignment were optimized for the best image quality. The scan speed was increased until it matched the electron beam scan raster speed. This allowed for easier and faster scanning. Next, the magnification was adjusted to around 12k. Several fields of view were obtained to evaluate silanization quality. For assessment of spore capture, the sample was moved to scan for spores from the top edge to the bottom edge of the sample. A total of ~54 mm^2^ were scanned for each chip. Any spores observed were imaged at higher magnification.

## 3. Results

**Figure 1:**
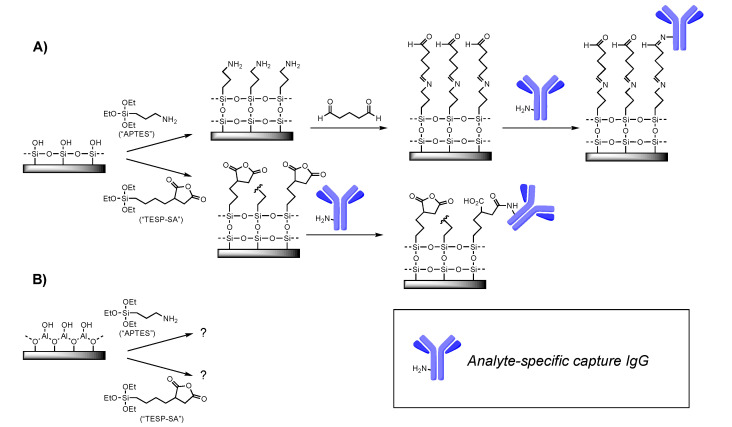
Surface chemistry tested. (**A**) Previous work from our lab and others has demonstrated the utility of APTES followed by glutaraldehyde, or TESP-SA, as reagents for depositing amine-reactive (electrophilic) functional groups on Si/SiO_2_ surfaces. (**B**) This work. For clarity, only one representative surface amine is shown on the IgG cartoon.

Covalent attachment of antibodies requires that the substrate carry a functional group able to react irreversibly with surface functionality on the antibody. Most commonly, this means providing an electrophile on the substrate, since nucleophilic amines as represented by lysine side chains are typically present on the antibody surface. Here, we tested two strategies: a one-step process in which deposition of TESP-SA would yield surface-bound anyhydrides, and the other a two-step process requiring silanization with APTES followed by reaction with glutaraldehyde (Figure 1). We and others have successfully used both silanes in the production of active sensors [22,23]. However, in addition to providing an operationally simpler approach, use of TESP-SA was expected to provide a more uniform reactive layer, given our past experience with these two silanes on Si/SiO_2_, and the known propensity of surface-bound glutaraldehyde to polymerize, forming a non-uniform, irregular surface [24].

**Figure 2:**
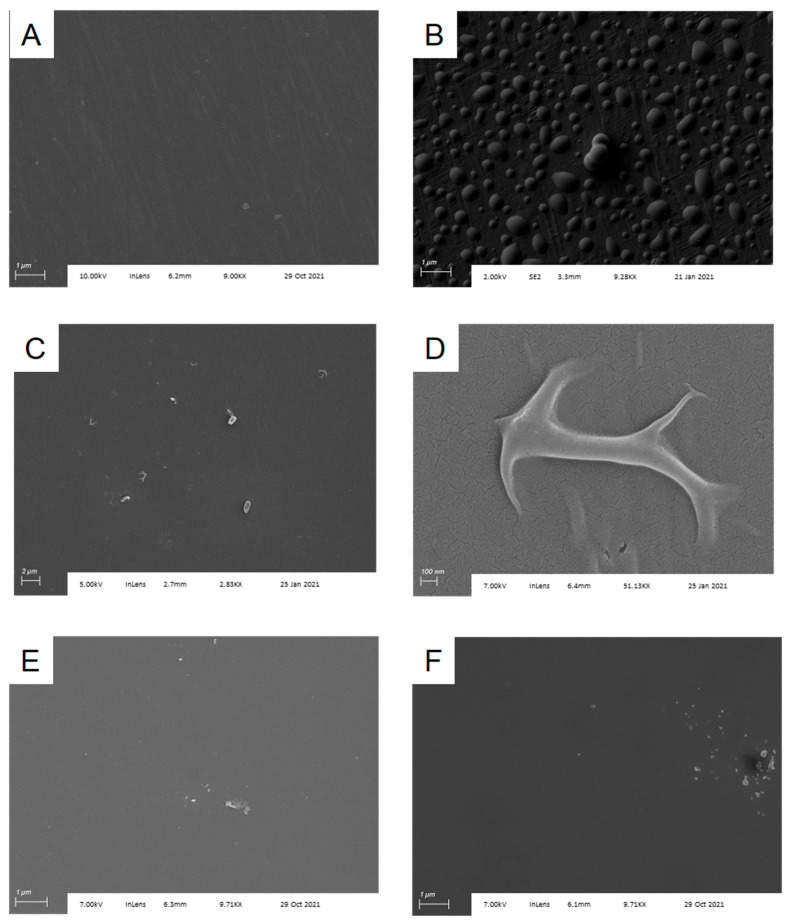
Silanization of Al/Al_2_O_3_ substrates. (**A**) Cleaned substrate as obtained from the supplier; (**B**) substrate following silanization with TESP-SA; (**C**) substrate following silanization with APTES; (**D**) higher magnification image of silanization “fold” from (**C**); (**E**) Si/SiO_2_ substrate following silanization with TESP-SA; (**F**) Si/SiO_2_ substrate following silanization with APTES.

Spectroscopic ellipsometry is one of the most common methods for examining thin film formation on substrates [25] and is a technique we have used extensively in the optimization of surface chemistry for Si/SiO_2_. However, spectroscopic ellipsometry relies on the availability of a well-validated model for fitting observed data. Lacking such a model for assessing silanized Al/Al_2_O_3_ substrates, we turned to SEM, as that would allow us to both observe silane-dependent changes in chip surface directly and also visualize individual *B. subtilis* spores during that phase of experiments. We immediately observed that TESP-SA behaved poorly on this surface, not producing a uniform film but rather yielding islands of deposited silane (Figure 2B). In contrast, APTES-functionalized chips had a much more uniform appearance, indicating either a complete lack of reaction, or formation of a contiguous film under the deposition conditions used (Figure 2C). As may be seen in Figure 2, APTES-functionalized surfaces were not pristine, however, with silane forming “folds” or aggregates in places. A close-up of this phenomenon is shown in Figure 2D. Since these structures were absent in SEM images of Al/Al_2_O_3_ substrates obtained from the manufacturer, we interpreted this as evidence of surface reaction. Given the known propensity of APTES to polymerize [19], it is likely that they are the result of localized APTES self-reaction. Reaction of Al/Al_2_O_3_ substrates with higher concentrations of APTES (5% or 10%) under analogous conditions produced greater amounts of surface aggregates (Supplementary Figure S1), leading us to use the 1% silane treatment for remaining studies. ATR-IR measurements of unfunctionalized and APTES-functionalized Al/Al_2_O_3_ substrates confirmed the presence of an -NH2 stretch in the APTES-functionalized material (Supplementary Figure S2). Finally, SEM images also confirmed that Si/SiO_2_ substrates were highly uniform in appearance following silanization with TESP-SA (Figure 2E) and APTES (Figure 2F), consistent with our previous work.

Surface hydrophilicity or hydrophobicity is an important component of successful sensor functionalization. To examine differences between Al/Al_2_O_3_ and Si/SiO_2_ before and after silanization, we measured water contact angles for each surface (Table 1). Both surfaces were sufficiently hydrophilic immediately after UV-ozone treatment that a contact angle could not be measured and remained hydrophilic (contact angle < 90°) throughout the process of silanization (for either APTES or TESP-SA) and glutaraldehyde treatment (after APTES). However, the Al_2_O_3_ surfaces were found to have contact angles that were between 12.7° and 22.4° higher (more hydrophobic) than the equivalent SiO_2_ surfaces.

**Figure 3:**
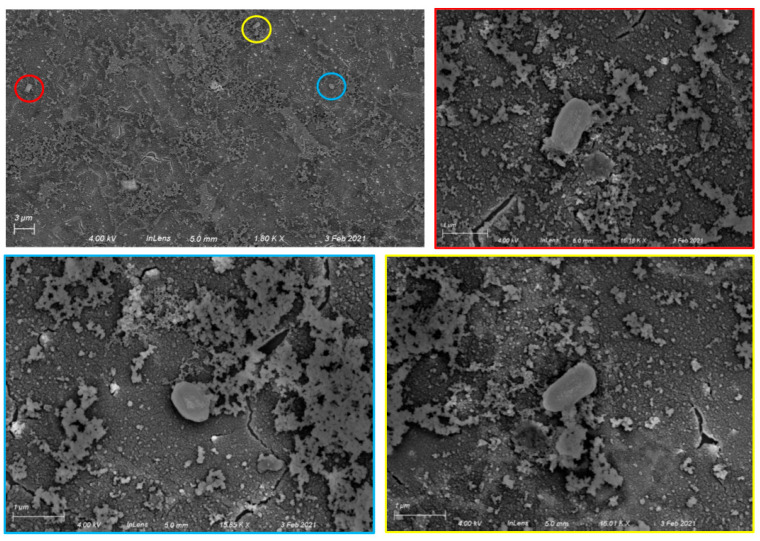
Specific capture of *Bacillus subtilis* spores on Al/Al_2_O_3_ surfaces with covalently attached anti-*B. subtilis* antibodies. **Top left**: 1800× image showing three spores (circled). **Top right and bottom**: higher magnification images (15,850×–16,180×) showing individual spores. The color framing each high-magnification image corresponds to the circled area in the lower-magnification image.

Having chosen APTES-silanized Al/Al_2_O_3_ substrates for further experiments, we then treated silanized substrates with aqueous glutaraldehyde followed by an anti-*B. subtilis* antibody to facilitate specific capture of *B. subtilis* spores. After a 2 h incubation with spore solution followed by water rinse, chips with a covalently attached anti-*B. subtilis* spore antibody captured approximately 64,000 spores per chip, based on observed densities of 3 spores per 3500 μm^2^ in multiple SEM images. Representative chip images are shown in Figure 3. Spores are readily distinguishable from surface debris (Figure S2) and have sizes and morphologies consistent with literature SEM data [26].

**Figure 4:**
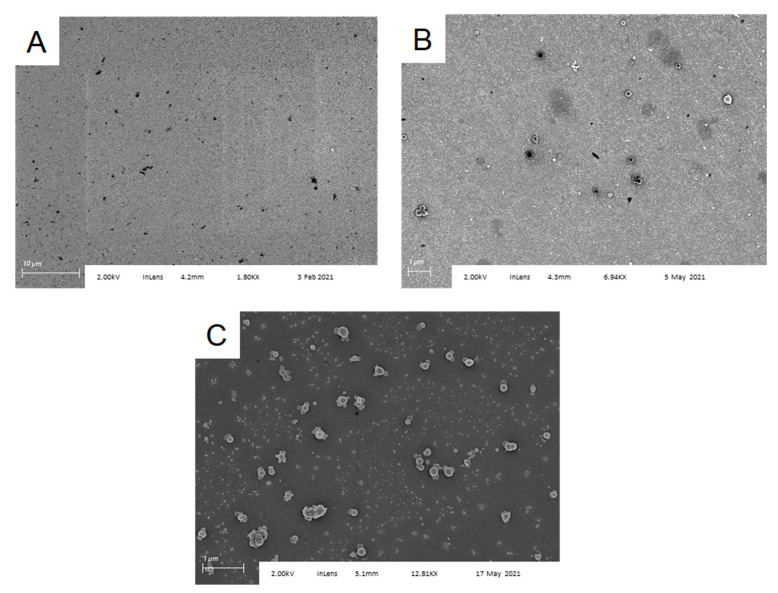
*Bacillus subtilis* spores are not captured by control surfaces functionalized with (**A**) APTES; (**B**) APTES followed by glutaraldehyde; and (**C**) APTES followed by glutaraldehyde, then anti-IL-6. Ambient dust or solution particulates coat the surface, but no spores are observed.

To control for nonspecific binding of spores to the chip surface, we also examined Al/Al_2_O_3_ chips functionalized only with APTES, APTES plus glutaraldehyde, or APTES plus glutaraldehyde chips onto which an anti-IL-6 antibody had been attached, after exposure to *B. subtilis* spores under identical conditions. SEM imaging of these three substrates (Figure 4) showed capture of various forms of dust and debris (likely part of the spore preparation), but no spores. Finally, *Bacillus atrophaeus* spores were not captured by anti-*B. subtilis* functionalized substrates (Figure S4). These results confirm specific spore capture by the anti-*B. subtilis* functionalized surfaces and indicate that the antibody retains both its activity and selectivity when immobilized.

## 4. Conclusions

We have successfully demonstrated that antibodies may be covalently attached to silanized, glutaraldehyde-treated ambient Al_2_O_3_ grown on an aluminum surface, and that these antibodies retain their ability to specifically bind a target of interest (here, *B. subtilis* spores). This result confirms that these substrates may be used effectively in the production of new biosensors. Silanization with APTES proceeded analogously to APTES silanization of Si/SiO_2_; however, reaction with TEPSA produced a highly nonuniform surface. This observation, coupled with the observation that functionalized Al/Al_2_O_3_ surfaces were consistently more hydrophobic than equivalent Si/SiO_2_ surfaces, despite both materials having high hydrophilicity prior to silanization, highlights the importance of carefully characterizing each step in the fabrication of sensors using new materials. While APTES deposited efficiently on the Al/Al_2_O_3_ surface, as noted above, it was not pristine. Vapor-phase silanization [11] would potentially produce a higher-quality silane film. We are currently investigating this possibility.

We were gratified to observe that Al/Al_2_O_3_ surfaces to which anti-*B. subtilis* antibodies were attached following silanization and glutaraldehyde treatment were capable of specific capture of *B. anthracis* spores. In contrast, substrates carrying anti-IL-6 antibodies, as well as substrates at each earlier step of the functionalization process (bare Al_2_O_3_, APTES, APTES + glutaraldehyde) did not capture spores. The lack of nonspecific capture on unfunctionalized, APTES, and APTES + glutaraldehyde substrates is likely assisted by the structure of the *B. subtilis* endospore [27]: the outer crust is heavily glycosylated [23] and therefore less susceptible to ionic interactions with the APTES amine group or covalent reaction with glutaraldehyde than a simple protein would be. In sum, these studies provide a starting point for future development of Al substrates carrying an ambient layer of Al_2_O_3_ as optical and electrical biosensors.

## Figures and Tables

**Table 1:** Contact angles measured for Si/SiO_2_ and Al/Al_2_O_3_ surfaces before and after reaction with TESP-SA silane or APTES silane followed by glutaraldehyde. All values are in degrees. “NM” = not measurable.

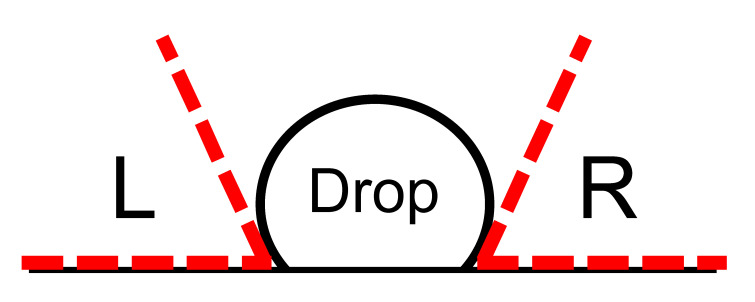	Drop 1		Drop 2		Drop 3			
Surface	L	R	L	R	L	R	Average	Standard Deviation
SiO_2_ Post UV-Ozone	NM	NM	NM	NM	NM	NM	NM	NM
SiO_2_ TESP-SA	53.5	56	53	51	54	58	54.3	2.2
SiO_2_ APTES	47	50	49	49	49	50	49.0	1.0
SIO_2_ APTES + Glutaraldehyde	34	36	35	35	35	37	35.3	0.9
Al_2_O_3_ Post UV-Ozone	NM	NM	NM	NM	NM	NM	NM	NM
Al_2_O_3_ TESP-SA	63	70	68	67	66	68	67.0	2.2
Al_2_O_3_ APTES	72.5	73	72	71	69	71	71.4	1.3
Al_2_O_3_ APTES + Glutaraldehyde	49	49	48	52	50	49	49.5	1.3

## Data Availability

All data is available from the corresponding author on request.

## Supplementary Materials

The following supporting information can be downloaded at: https://www.mdpi.com/article/10.3390/s22093441/s1, Figure S1: Comparison of silanization with 1%, 5%, and 10% solutions of APTES in dry toluene. Significantly greater numbers of aggregates are observed in substrates treated with the 5% or 10% solutions. Arrows highlight representative aggregates, with their measured size; Figure S2: ATR-IR spectrum of APTES-functionalized Al/Al_2_O_3_ substrate. A band for the APTES-NH_2_ group is readily visible at 3240 cm^−1^; this band is not visible in an unfunctionalized Al/Al_2_O_3_ substrate; Figure S3: Spores have a distinct size and shape, and are readily distinguished from debris on the surface of the chip; Figure S4: Al/Al_2_O_3_ substrate functionalized with an anti-*Bacillus subtilis* antibody does not capture *Bacillus atrophaeus* spores, confirming specificity.
