# Supplementary material for: Development of Methods for Specific Capture of Biological Targets on Aluminum Substrates: Application to Bacillus subtilis Spore Detection as a Model for Anthrax
Source: Sensors (Basel). 2022 Apr 30;22(9):3441. doi: 10.3390/s22093441 (PMC9106032; doi:10.3390/s22093441)

Development of methods for specific capture of biological targets on aluminum substrates: application to *Bacillus subtilis* spore detection as a model for anthrax.

Ethan P. Luta and Benjamin L. Miller*

Department of Dermatology, University of Rochester, Rochester, NY 14642

SUPPLEMENTARY INFORMATION

Supplementary Figure S1: Comparison of silanization with 1%, 5%, and 10% solutions of APTES in dry toluene. Significantly greater numbers of aggregates are observed in substrates treated with the 5% or 10% solutions. Arrows highlight representative aggregates, with their measured size.

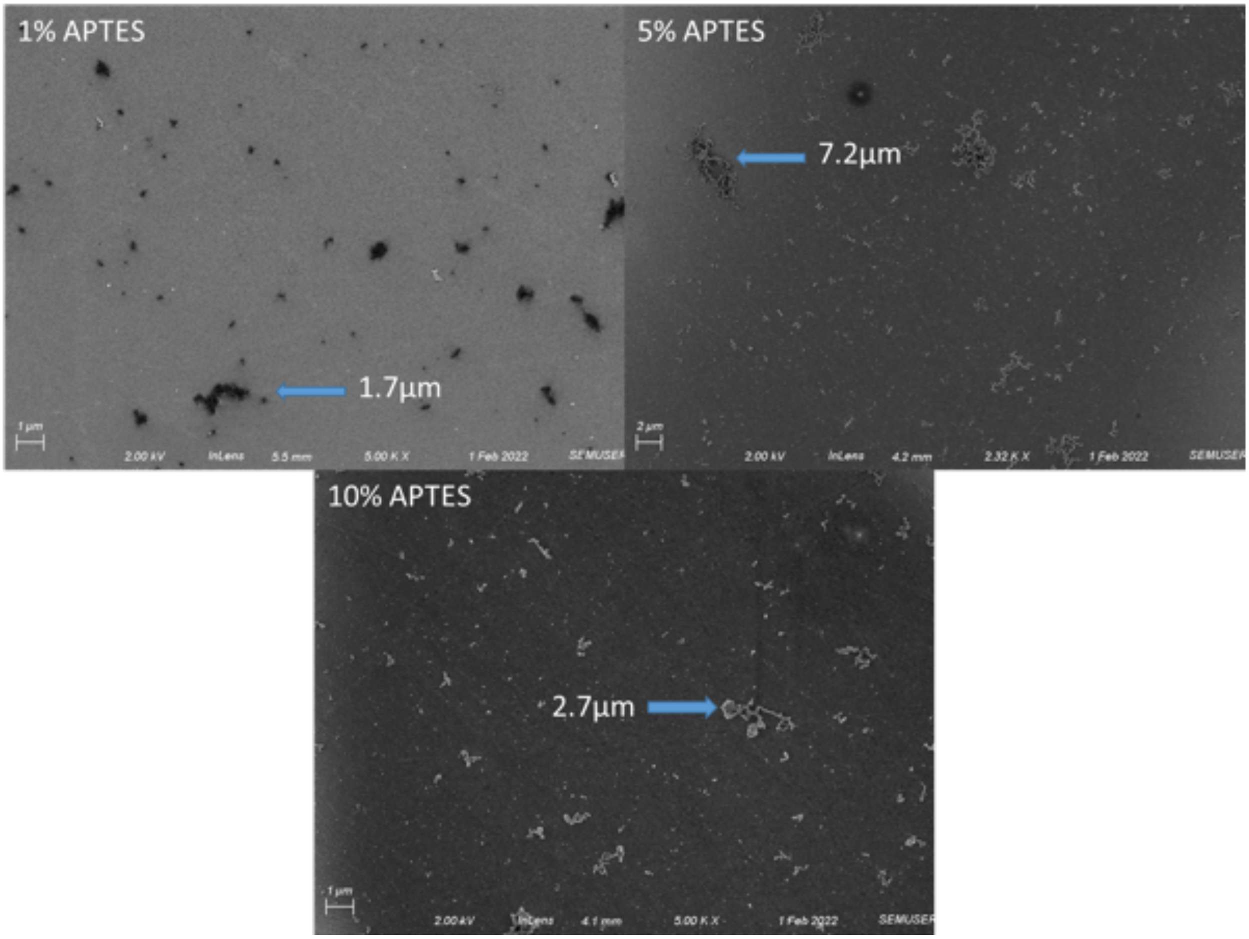

Figure S2: ATR-IR spectrum of APTES-functionalized Al/Al_2O_3 substrate. A band for the APTES $-NH_2$ group is readily visible at 3240 cm^{-1}; this band is not visible in an unfunctionalized Al/Al_2O_3 substrate.

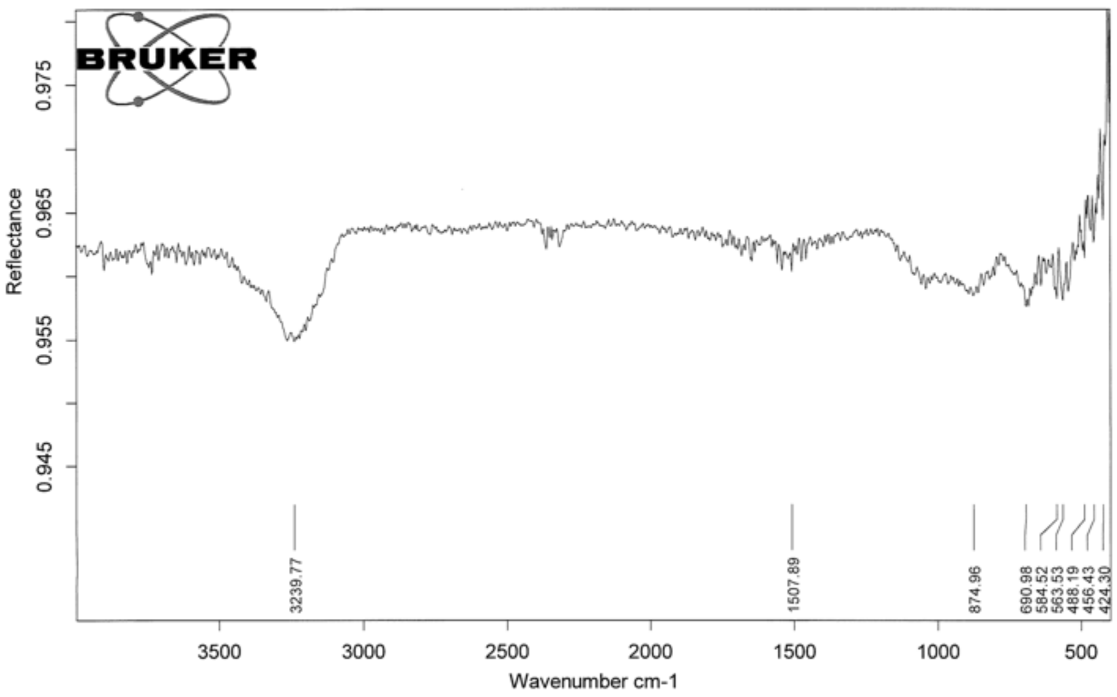

Figure S3: Spores have a distinct size and shape, and are readily distinguished from debris on the surface of the chip.

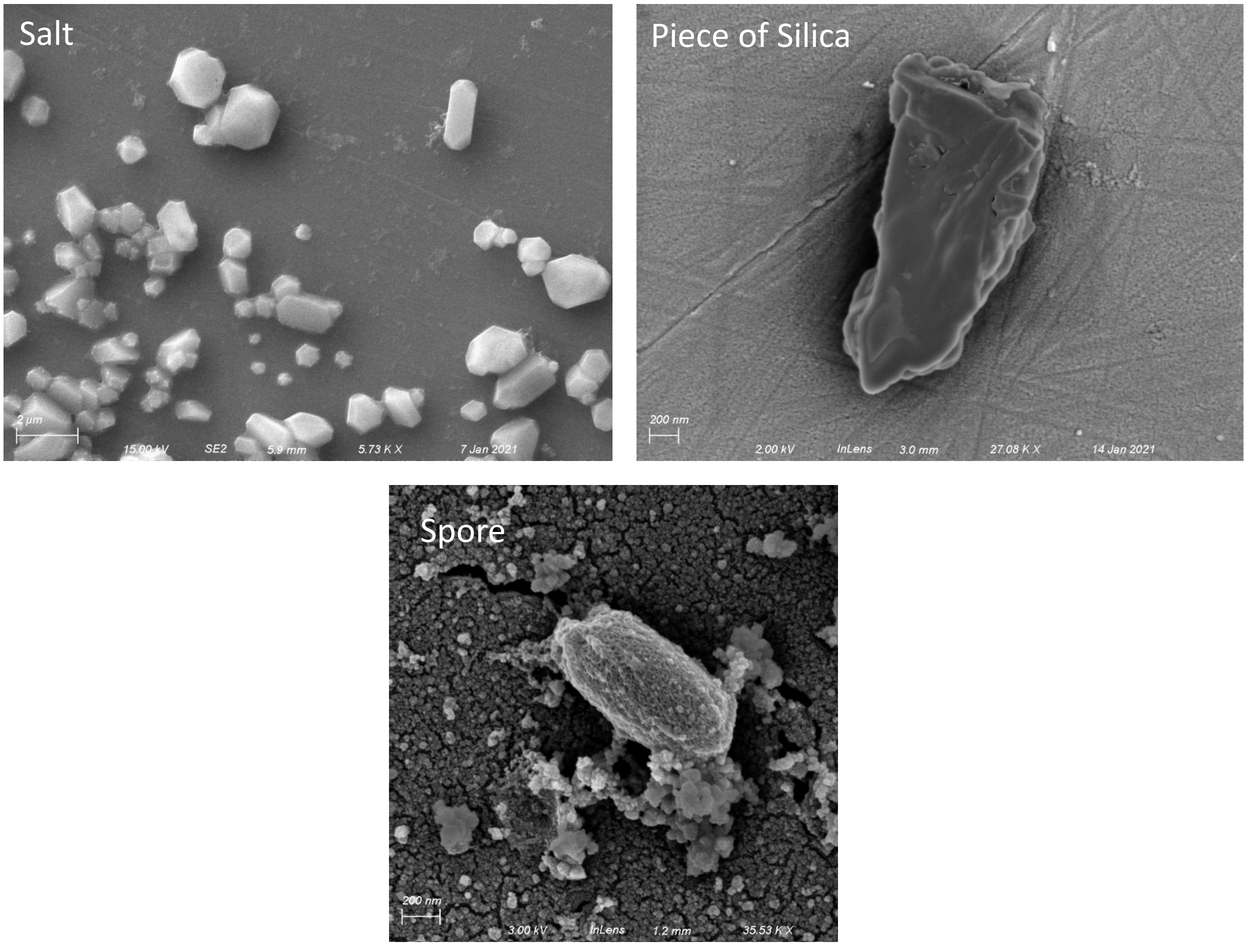

Figure S4: Al/Al_2O_3 substrate functionalized with an anti-*Bacillus subtilis* antibody does not capture *Bacillus atrophaeus* spores, confirming specificity.

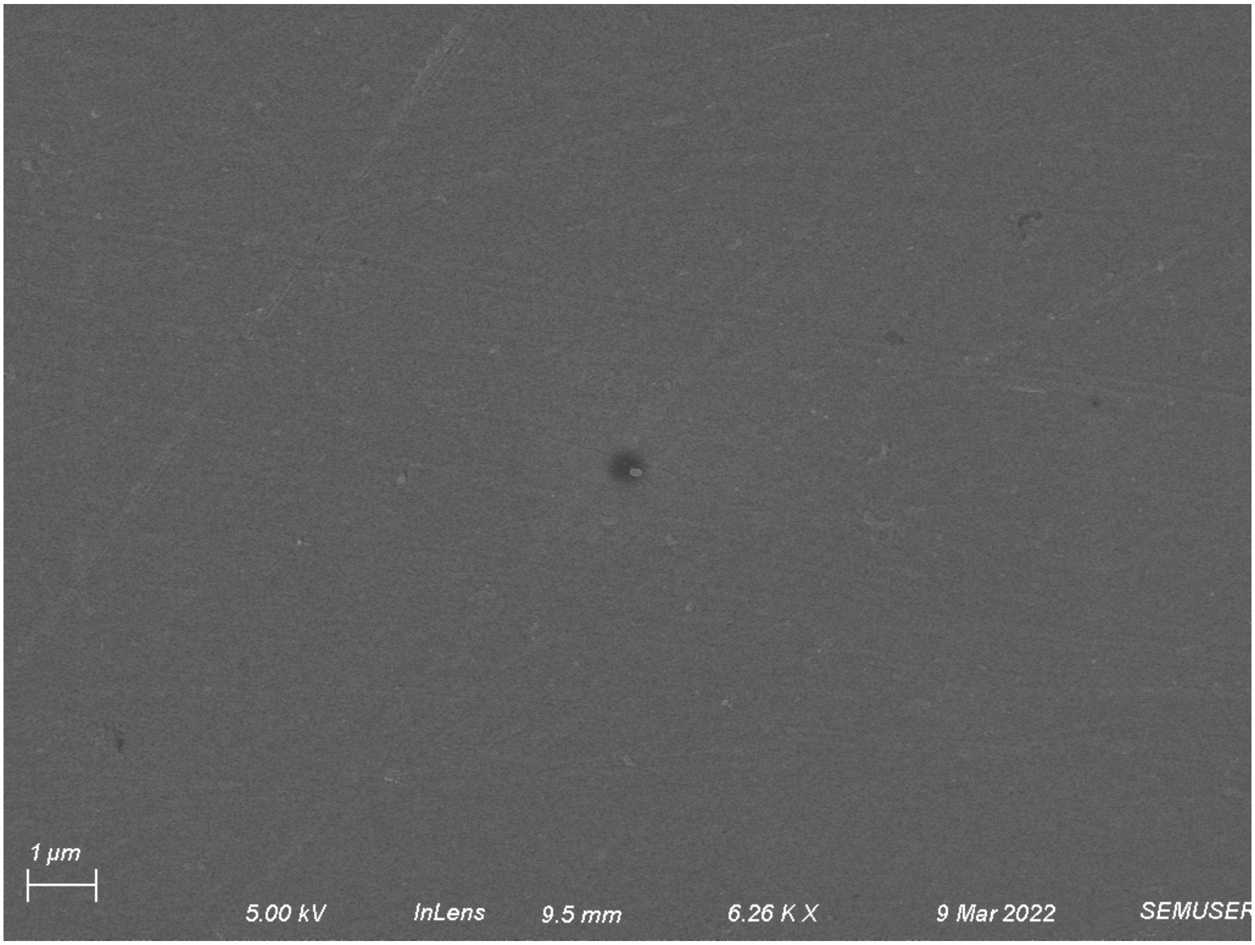

Supplement: Supplementary file 1 [file sensors-22-03441-s001.zip › sensors-1564868-supplementary.pdf]
